# Diagnostic Value of DECT-Based Collagen Mapping for Assessing the Distal Tibiofibular Syndesmosis in Patients with Acute Trauma

**DOI:** 10.3390/diagnostics13030533

**Published:** 2023-02-01

**Authors:** Leon David Gruenewald, Daniel H. Leitner, Vitali Koch, Simon S. Martin, Ibrahim Yel, Scherwin Mahmoudi, Simon Bernatz, Katrin Eichler, Tatjana Gruber-Rouh, Daniel Pinto Dos Santos, Tommaso D’Angelo, Thomas J. Vogl, Christian Booz

**Affiliations:** 1 Division of Experimental Imaging, Department of Diagnostic and Interventional Radiology, University Hospital Frankfurt, 60590 Frankfurt am Main, Germany; 2 Department of Diagnostic and Interventional Radiology, University Hospital Frankfurt, 60590 Frankfurt am Main, Germany; 3 Diagnostic and Interventional Radiology Unit, BIOMORF Department, University Hospital Messina, 98124 Messina, Italy; 4 Department of Radiology and Nuclear Medicine, Erasmus MC, 3015 GD Rotterdam, The Netherlands

**Keywords:** CT-dual energy computed tomography, ankle joint, syndesmosis, collagen

## Abstract

**Background**: Injury to the distal tibiofibular syndesmosis (DTFS) is common in patients with trauma to the ankle, but diagnostic accuracy of conventional X-ray and CT is insufficient. A novel dual energy CT (DECT) post-processing algorithm enables color-coded mapping of collagenous structures, which can be utilized to assess the integrity of the DTFS. **Methods**: Patients were included in this retrospective study if they underwent third-generation dual-source DECT followed by 3T-MRI or ankle joint surgery within 14 days between January 2016 and December 2021. Three radiologists blinded to all patient data independently evaluated grayscale images and, after 8 weeks, grayscale and collagen mapping images for the presence of ligamentous injury or avulsion fractures of the DTFS. MRI and surgery provided the reference standard. Diagnostic accuracy parameters were calculated for all ratings, and a comparison of ROC curve analysis was performed to evaluate the incremental diagnostic value of color-coded images over grayscale images. **Results**: A total of 49 patients (median age 49 years; 32 males) were evaluated. Application of collagen mapping significantly increased sensitivity (25/30 [83%] vs. 20/30 [67%]), specificity (110/118 [93%] vs. 70/118 [60%]), positive predictive value (25/33 [76%] vs. 20/67 [30%]), negative predictive value (110/115 [96%] vs. 70/80 [88%]), and accuracy (134/147 [91%] vs. 90/147 [61%]) for the detection of injury to the DTFS (all parameters, *p* < 0.001). Collagen mapping achieved higher diagnostic confidence, image quality, and noise scores compared to grayscale CT (all parameters, *p* < 0.001). **Conclusions**: Collagen mapping yields substantially higher diagnostic accuracy and confidence for assessing the integrity of the distal tibiofibular syndesmosis compared to grayscale CT in patients with acute trauma. The application of this algorithm can accelerate the adequate diagnosis and treatment of DTFS injury in clinical routine.

## 1. Introduction

The collateral ligaments and the distal tibiofibular syndesmosis (DTFS) are major stabilizers of the ankle joint and are frequently injured in athletes. The DTFS comprises four ligaments and two bones; syndesmotic injury, however, usually refers to an injury to one of the ligamentous structures: the anterior tibiofibular ligament (ATIFL), the posterior tibiofibular ligament (PTIFL), the transverse ligament, and the interosseous ligament [1].

Injury to the collateral ligaments is common, but surgical intervention is usually not required because scarring re-establishes sufficient stability. For syndesmotic injury, incidences of up to 18% have been reported in patients with an ankle sprain. In contrast to an injury to the collateral ligaments, missed syndesmotic injury can result in functional limitations and possible ankle dysfunction if adequate treatment is delayed [2,3].

Following trauma, clinical examination and radiographs are regularly performed. However, the accuracy of clinical tests and radiographic examination for assessing DTFS injury is insufficient, and MRI remains the reference standard for imaging syndesmotic injury [4,5,6]. Ankle sprains commonly occur during on-call times, which limits the availability of MRI and can delay adequate diagnosis and treatment. CT is readily available to assess musculoskeletal trauma and is frequently performed when conventional imaging is inconclusive, or a more detailed depiction of the osseous structures and the joint surface is required to aid clinical and surgical management. Extracting information regarding the integrity of ligamentous structures from routinely performed CT examinations could therefore accelerate the diagnosis and initiation of adequate treatment for ligamentous injury of the DTFS or serve as a diagnostic means to rule out ligamentous injury.

Single-energy CT can detect bone fragments, minor diastasis, and subluxation but provides insufficient resolution and soft tissue contrast to depict injury to the DTFS [7]. Dual-energy CT (DECT) facilitates material differentiation, which increases the quality of soft tissue visualization and provides novel information for different musculoskeletal applications [8,9,10,11,12,13]. Third-generation dual-source DECT further improved both the spatial resolution and the capacity for material differentiation, with advanced possibilities for assessing soft tissue structures. In this context, post-processing algorithms have been developed to visualize collagenous structures by quantification of the heavy sidechains of hydroxyproline and hydroxylysine [14,15]. However, these algorithms have not yet been investigated for the DTFS.

Therefore, this study aimed to evaluate the diagnostic accuracy, diagnostic confidence, and overall image quality of third-generation dual-source DECT color-coded mapping of collagenous structures to assess the distal tibiofibular syndesmosis in comparison to standard grayscale CT. 

## 2. Materials and Methods

This is a single center, retrospective study. The protocol of the study was approved by the ethics committee of the University Hospital Frankfurt (approval number 20-1054, amendment number 3) and complied with the Declaration of Helsinki. The approval included the retrospective gathering of patient demographics, clinical data, and imaging data, as well as the conduction of phone interviews to inquire about pre-existing injuries or injuries diagnosed by external hospitals or physicians. Informed consent to utilize the data of the individual patient was waived. The data presented are fully anonymized.

### 2.1. Patient Selection

Sixty-five patients with acute injury to the ankle joint who underwent unenhanced third-generation dual-source DECT between January 2016 and December 2021, followed by supplementary MRI and/or surgical inspection of the ankle joint within 14 days, were considered for study inclusion. Patients with inadequate imaging quality and metal artifacts were excluded from this study. 

### 2.2. CT Protocol

CT images were acquired on a third-generation dual-source CT system (SOMATOM Force; Siemens Healthineers, München, Germany) in which two X-ray tubes operated at different kV settings (tube 1: 90 kVp, 180 mAs; tube 2: Sn150 kVp; 0.64 mm tin filter, 180 mAs). No contrast agent was administered during the examinations. In each CT examination, three image sets were acquired: 90 kVp, Sn150 kVp, and weighted average (ratio, 0.5:0.5). Image series in all planes (section thickness 1 mm, increment 0.75 mm) were reconstructed with dedicated dual-energy bones (Br69f) and soft-tissue kernels (Br40). 

### 2.3. CT Post-Processing

Color-coded collagen mapping was performed on a Siemens *syngo.via* VB50 (Siemens Healthineers, München, Germany) with the following default settings, as recommended by the vendor. 

Application profile: Knee. Settings: Collagen. Public Layout: Fat Map. Width: 65. Level: −30.

Color-coded reconstructions in three planes (section thickness 1 mm, increment 0.75 mm) were sent to the PACS (GE Healthcare, Germany). The duration for each reconstruction was measured from launching Siemens *syngo.via* until image export to the PACS system was initiated. All color-coded reconstructions were created with the same version of the provided software and identical imaging protocols.

### 2.4. MRI Protocol

MRI examinations were performed on a 3-Tesla system (PrismaFit, Siemens Healthineers, München, Germany). All studies included axial unenhanced T1-weighted turbo spin-echo sequences with and without fat suppression and proton density-weighted sequences with and without fat suppression in all three planes (ST: 3 mm). The pulse sequence parameters (repetition time, echo time, flip angle), FOV, and acquisition matrix were adapted for every examination.

### 2.5. Image Analysis

Image analysis was performed using a PACS workstation from the clinical routine (Centricity 7.0; GE Healthcare, Germany). The preset windows of the workstation could be freely modified.

To provide an independent reference standard, two board-certified radiologists (K.E. and T.V.) with 18 and 36 years of experience in musculoskeletal imaging independently performed a reading of all MRI series. In case of disagreement (n = 3), a third board-certified radiologist (T.G.) with 12 years in musculoskeletal imaging was consulted. Reported is the majority decision.

A consecutive reading of all CT images was independently performed by three different radiologists (two board-certified radiologists (C.B. and I.Y.) and one radiologist in training (V.K.) with 3 to 8 years of experience in musculoskeletal imaging. All readers were blinded to clinical data, previous imaging, and follow-up examinations. 

Two protocols were provided for the assessment of the DTFS: protocol 1 = standard grayscale images in all three planes and protocol 2 = standard grayscale images and color-coded reconstructions in all three planes, as described above. A time interval of 8 weeks was kept between readout sessions to reduce observer recall bias. All readers reviewed both protocols for the presence of injury to the DTFS in an intention-to-treat approach (1 = rupture absent, 2 = rupture present, 3 = avulsion). 

Moreover, readers rated their overall diagnostic confidence in assessing DTFS integrity, as well as the image noise and the image quality (ranging from 1 = poor to 5 = excellent) for each imaging protocol and patient.

### 2.6. Surgical Inspection

The surgical inspection was performed during routine surgical management by two board-certified orthopedic surgeons (V.H. and K.Z.) with 6 and 11 years of experience. When MRI and surgical inspection were available, the surgical inspection was used as the reference standard.

### 2.7. Statistical Analysis

Statistical analysis was performed with commercial statistic software (Prism 9 for macOS, version 9.0.1, GraphPad Software LLC, San Diego; MedCalc for Windows, Version 20.022, MedCalc, Mariakerke, Belgium). Differences in baseline characteristics were assessed using *t*-tests, if applicable, or chi-squared tests. Inter-reader agreement was evaluated by computing weighted Fleiss’ κ.

Imaging findings were analyzed individually for each type of injury to the DTFS, as mentioned above. A second analysis was performed after lesions were dichotomized (0 = injury absent, 1 = injury present). All findings were compiled in cross-tables. Diagnostic accuracy parameters (sensitivity, specificity, positive predictive value (PPV), negative predictive value (NPV), and area under the curve (AUC)) for the detection of injury to the DTFS were calculated. A receiver operator characteristic (ROC) curve comparison was used to determine the additional diagnostic value of color-coded collagen mapping over grayscale imaging. Statistical significance was given if *p* < 0.05.

## 3. Results

**Figure 1:**
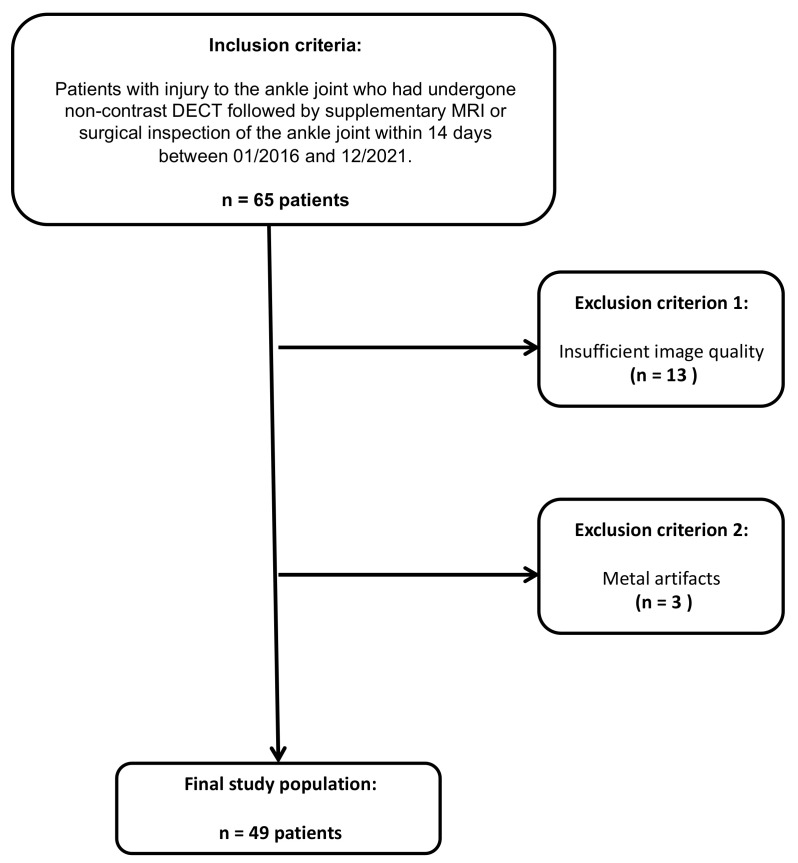
STARD (standards for the reporting of diagnostic accuracy studies) flow chart of patient inclusion.

**Figure 2:**
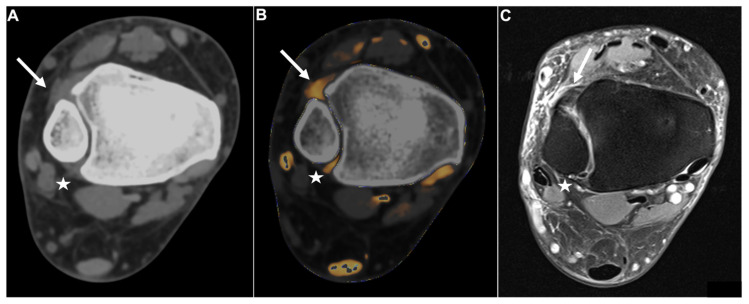
Intact DTFS. Standard axial unenhanced grayscale CT (**A**), color-coded collagen reconstructions (**B**), and proton density-weighted unenhanced MRI series with fat saturation (**C**) of a patient with an ankle sprain. No diagnostic assessment of the DTFS is possible in the grayscale images. After collagen mapping, the ATIFL (**arrow**) and PTIFL (**star**) demonstrate strong collagen signals, indicating that they are not injured. MRI confirmed the integrity of both ligaments. Abbreviations: DTFS, distal tibiofibular syndesmosis. ATIFL, anterior tibiofibular ligament. PTIFL, posterior tibiofibular ligament.

Of the sixty-five patients considered for study inclusion, thirteen were excluded due to inadequate imaging quality, and three patients were excluded due to metal artifacts. Ultimately, 49 patients who had undergone unenhanced third-generation dual-source DECT of the ankle joint followed by MRI or surgical inspection were included (32 male and 17 female, median age, 49 years, IQR 33–60 years) (Figure 1). The reference standard revealed an injury to the DTFS in 10 patients (20.4 %; complete tear = 5; avulsion = 5). No demographic differences were observed between patients with and without injury to the DTFS (Table 1). The mean interval between dual-energy CT and MRI or surgical inspection was seven days (range, 0–12 days). Color-coded collagen mapping required an average of 4 min (range, 1–7 min). An example case without injury to the ATIFL is given in Figure 2.

No significant differences were observed between the demographics of patients with injury to the DTFS and patients without injury. 

### 3.1. Diagnostic Accuracy of DTFS Injury

**Figure 3:**
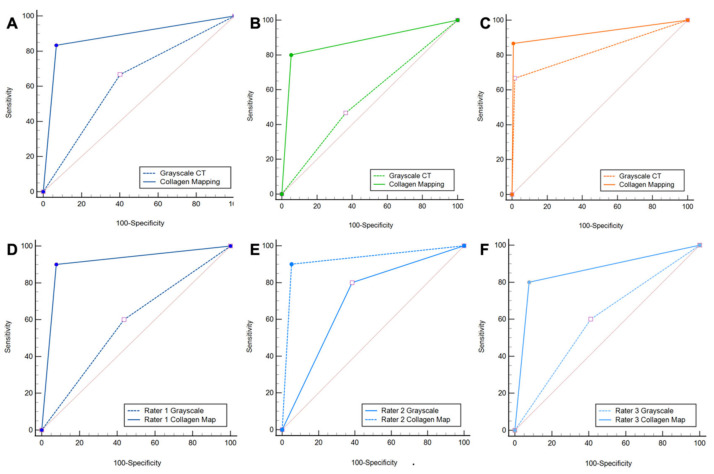
ROC curve analysis. ROC curve analysis shows increased diagnostic accuracy of protocol 2, which included color-coded collagen mappings and grayscale CT (solid line) over protocol 1, which included only standard grayscale CT (dotted line) for the depiction of DTFS injury (**A**)**.** Subgroup analysis confirmed the increased diagnostic accuracy for ligamentous tears (**B**), but not for avulsion fractures (**C**). All three readers improved significantly when color-coded collagen mapping was available (**D**–**F**). Abbreviations: ROC, receiver operating characteristic.

Protocol 2, which includes standard grayscale CT images and color-coded collagen maps, showed higher overall sensitivity (25/30 [83%] vs. 20/30 [67%]), specificity (110/118 [93%] vs. 70/118 [60%]), PPV (25/33 [76%] vs. 20/67 [30%]), NPV (110/115 [96%] vs. 70/80 [88%]), and accuracy (134/147 [91%] vs. 90/147 [61%]) for the detection of injury to the DTFS compared to protocol 1 (all parameters, *p* < 0.001, ΔAUC = 0.25) (Table 2). Increases in AUC between protocol 1 and protocol 2 were significant for ligamentous tears (0.87 vs. 0.55, ΔAUC = 0.32, *p* < 0.001); however, no significant increase in diagnostic accuracy was observed for avulsions of the DTFS (0.93 vs. 0.83, ΔAUC = 0.10, *p* =.15) (Figure 3).

**Figure 4:**
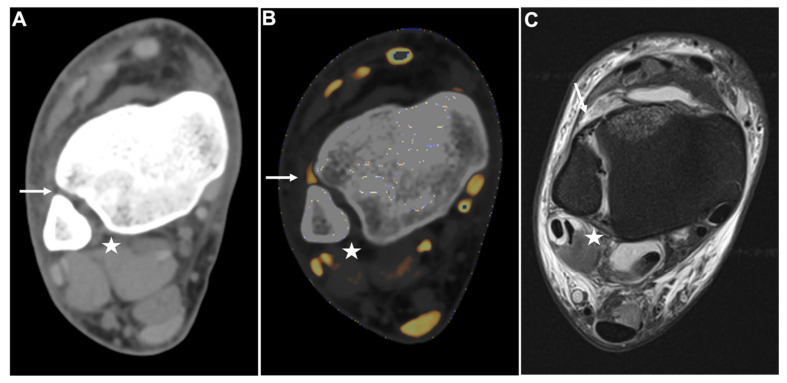
PTIFL strain. Standard axial unenhanced grayscale CT (**A**), color-coded collagen reconstructions (**B**), and proton density-weighted unenhanced MRI series with fat saturation (**C**) of a patient with an ankle sprain. While the ATIFL (**arrow**) and PTIFL (**star**) are visible in the grayscale images, no diagnostic assessment of both ligaments is accurately possible. After collagen mapping, the ATIFL is visible and intact (**arrow**)**.** However, no collagen signal can be acquired for the PTIFL (**star**), suggestive of rupture. MRI images show an intact ATIFL (**arrow**) and a partial tear of the PTIFL (**star**), which likely caused a drop of the collagen content below acquirable thresholds. Abbreviations: ATIFL, anterior tibiofibular ligament. PTIFL, posterior tibiofibular ligament.

**Figure 5:**
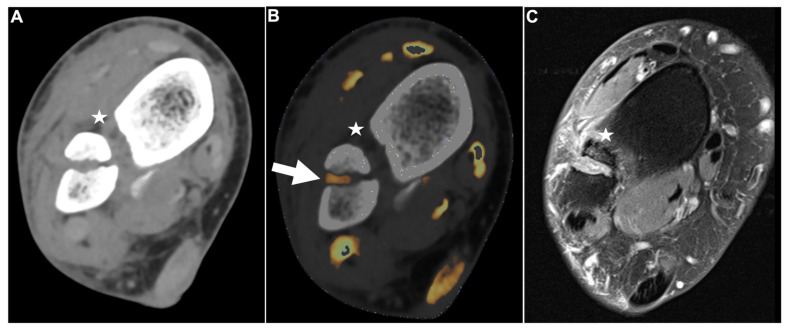
Fractured fibula with DTFS tear. Standard axial unenhanced grayscale CT (**A**), color-coded collagen reconstructions (**B**), and proton density-weighted unenhanced MRI series with fat saturation (**C**) of a fractured fibula with rupture of the ATIFL (**stars**). Notably, in the grayscale images, the ATIFL is visible (**star**); however, collagen mapping reveals no collagen signal, indicating a rupture. Note the distortion of the collagen signal by bleeding in the fractured region (**B, arrow**). MRI confirmed rupture of the ATIFL (**star**) and PTIFL (not pictured due to angulation). Abbreviations: ATIFL, anterior tibiofibular ligament. PTIFL, posterior tibiofibular ligament.

All readers improved significantly after the readout of protocol 2. The least experienced radiologist showed the most remarkable improvement in diagnostic accuracy for the detection of DTFS injury compared to more experienced readers with higher overall sensitivity (9/10 [90%] vs. 6/10 [60%]), specificity (36/39 [92%] vs. 22/39 [56%]), PPV (9/12 [75%] vs. 6/23 [26%]), NPV (36/37 [97%] vs. 22/26 [85%]), and accuracy (45/49 [92%] vs. 28/49 [57%], all comparisons, *p* < 0.001) (Table 3). Furthermore, the availability of collagen maps increased inter-reader agreement (κ = 0.62 for protocol 1 and κ = 0.72 for protocol 2). Example cases demonstrating improvement in the detection of DTFS lesions are illustrated in Figure 4 and Figure 5.

### 3.2. Diagnostic Confidence, Image Quality, and Image Noise

The diagnostic confidence of all readers for detecting injuries to the DTFS increased significantly when color-coded collagen maps were included in the evaluation protocol (4.1 ± 0.8 vs. 2.3 ± 1.0, *p* < 0.001). Inter-reader agreement was low for protocol 1 (κ = 0.33) and good for protocol 2 (κ = 0.71). No significant differences were observed regarding the diagnostic confidence between less and more experienced readers (Table 4).

Grayscale images received a mean rating of 2.4 ± 0.9 for image quality compared to 3.9 ± 0.7 for color-coded collagen images (*p* < 0.001). Inter-reader agreement significantly increased when color-coded collagen maps were included in the evaluation protocol (κ = 0.32 for grayscale, κ = 0.70 for color-coding).

Image noise was perceived lower in color-coded images compared to grayscale images (3.6 ± 0.6 vs. 2.3 ± 1.0, *p* < 0.001), but the inter-reader agreement was only moderate for both protocols (κ = 0.36 for grayscale, κ = 0.40 for color-coding).

## 4. Discussion

In this study, we demonstrate the diagnostic utility of a novel DECT post-processing algorithm for the color-coded depiction of the DTFS and the evaluation for syndesmotic injury. The availability of color-coded collagen maps significantly increased the diagnostic accuracy (134/147 [91%] vs. 90/147 [61%]) and diagnostic confidence (4.1 ± 0.8 vs. 2.3 ± 1.0) of all readers for the assessment of DTFS injury (all measurements, *p* < 0.001). Inter-reader agreement, perceived image quality, and perceived image noise also showed significant improvements, which suggests greater reliability of color-coded collagen maps (all measurements, *p* < 0.001).

Due to fast acquisition times and high spatial resolution, CT remains an invaluable tool for fracture assessment. However, insufficient soft tissue contrast of conventional multi-detector CT (MDCT) does not allow for an accurate evaluation of ligamentous structures, which might be required following acute trauma [16]. While DECT is not an entirely new technology, the increase in spatial resolution of third-generation dual-source DECT devices together with an improved possibility for material decomposition allows for a more accurate evaluation of structures that could not be visualized before. In line with this, recent studies demonstrated improved material decomposition and higher spatial resolution of third-generation dual-source DECT, which enable more accurate identification and depiction of the hydroxylysine and hydroxyproline side chains contained in collagen molecules [13,14,15,17,18]. However, no studies have evaluated the potential of color-coded collagen maps for assessing a syndesmotic injury.

The high specificity and NPV underline the potential of DECT to rule out fractures and ligamentous injuries with only one examination, for example in patients with inconspicuous conventional imaging but persistent symptoms of pain or instability. Additional DECT applications utilizing material differentiation, such as the visualization of bone marrow edema to identify acute fractures, further emphasize this role [15,19]. For specific clinical situations, DECT could, therefore, emerge as an alternative imaging modality in circumstances where MRI is not available or not feasible. The reconstruction of color-coded collagen maps took four minutes on average and is, therefore, possible in daily clinical practice.

For the identification of avulsion fractures, only a moderate and statistically non-significant increase in diagnostic accuracy was demonstrated in our study using color-coded collagen reconstructions. Since a dedicated bone kernel, the gold standard for fracture assessment, was included both in protocol 1 and protocol 2, this result was expected. The slight increase in diagnostic accuracy could, however, be attributed to the fact that color-coded reconstructions allow the assessment of the ligamentous integrity of the DTFS in avulsion fractures and thereby help the discrimination of different types of injury. 

This retrospective study has some limitations we want to mention. First, with only forty-nine patients and one device, the study population and generated pictures are limited, and further evaluation with multiple devices and larger patient cohorts are required in the future. Second, compared to other collagenous tissue, such as the cruciate ligaments, the components of the DTFS have relatively low collagen content. This sometimes resulted in a drop of the collagen signal below the lower threshold for detection when only a partial tear was present for patients with lesions to the ATIFL and PTIFL, causing the ligaments to disappear on the collagen mapping reconstructions. Therefore, the disappearance of the ATIFL and PTIFL in collagen mapping does not prove rupture, but can also occur in partial tears. Third, because patients with fractures that underwent surgical management did not receive MRI before surgery, we adopted a mixed reference standard including MRI and surgical inspection. Fourth, we evaluated the diagnostic value of color-coded collagen maps derived from third-generation dual-source DECT compared to grayscale images generated on the same device. Due to the high spatial resolution and material differentiation, the soft-tissue contrast of these grayscale images is superior to standard MDCT grayscale images, and the actual diagnostic value of collagen mapping over single-energy CT could be underestimated from the results of this study. Fifth, this technology is only available for DECT devices, since MDCT does not generate enough spatial information for different energy levels to allow for material decomposition. Last, the color-coded collagen mapping algorithm we utilized is vendor specific. Although all major vendors offer dual-energy CT, the results of our study can, therefore, not be generalized.

In conclusion, we demonstrate that collagen maps derived from third-generation dual-source DECT of the DTFS significantly improve the diagnostic accuracy and diagnostic confidence of reading radiologists for evaluating syndesmotic injury compared to standard grayscale CT in patients with acute trauma.

## 5. Conclusions

Color-coded collagen maps derived from dual-energy CT yield higher diagnostic accuracy and higher diagnostic confidence for the detection and exclusion of injury to the DTFS compared to standard grayscale CT.

## Figures and Tables

**Table 1:** Characterization of the patient population.

Variables, n (%) or Median (IQR)	Total (n = 49)	DTFS Injury (n = 10)	W/o DTFS Injury (n = 39)	*p*-Value
Age (years)	49 (33–60)	53 (43–61)	48 (32–60)	0.46
Sex (n)				0.55
Male	32 (65.3 %)	7 (70 %)	25 (64.1 %)	
Female	17 (34.7 %)	3 (30 %)	14 (35.9 %)	

Abbreviations: IQR, interquartile range. DTSF, distal tibiofibular syndesmosis; W/o, without. Age is given ± standard deviation (SD). Age is given as the median (IQR).

**Table 2:** Diagnostic accuracy of standard grayscale CT and color-coded collagen mapping for syndesmotic injury.

Variables, n (%) [95% Confidence Interval]	Sensitivity	Specificity	PPV	NPV	Accuracy	AUC	*p*-Value
Total							
Protocol 1	20/30 (67%) [47–83%]	70/118 (60%) [50–69%]	20/67 (30%) [23–37%]	70/80 (88%) [81–92%]	90/147 (61%) [53–69%]	0.63 [0.55–0.71]	<0.001
Protocol 2	25/30 (83%) [65–94%]	110/118 (93%) [87–97%]	25/33 (76%) [61–86%]	110/115 (96%) [91–95%]	134/147 (91%) [85–95%]	0.88 [0.82–0.93]	<0.001
Ligamentous Tear							
Protocol 1	7/15 (47%) [21–73%]	84/132 (64%) [55–72%]	7/55 (13%) [8–21%]	84/92 (91%) [87–95%]	91/147 (62%) [54–70%]	0.55 [0.47–0.63]	<0.001
Protocol 2	12/15 (80%) [52–96%]	125/132 (95%) [89–98%]	12/19 (63%) [44–78%]	125/128 (98%) [94–99%]	137/147 (93%) [88–97%]	0.87 [0.81–0.92]	<0.001
Bony Avulsion							
Protocol 1	10/15 (67%) [39–88%]	130/132 (99%) [95–100%]	10/12 (83%) [55–95%]	130/135 (96%) [93–98%]	140/147 (95%) [90–98%]	0.83 [0.76–0.88]	0.15
Protocol 2	13/15 (87%) [60–98%]	131/132 (99%) [96–100%]	13/14 (93%) [65–99%]	131/133 (99%) [95–100%]	144/147 (98%) [94–100%]	0.93 [0.88–0.97]	0.15

Abbreviations: PPV: positive predictive value, NPV: negative predictive value, AUC: area under the curve. Numbers in square brackets are confidence intervals. Diagnostic accuracy of protocol 1 (standard grayscale CT) and protocol 2 (standard grayscale CT + color-coded collagen maps) for assessing DTFS injury. MRI or surgical inspection provided the standard of reference.

**Table 3:** Individual readings of diagnostic accuracy.

Variables, n (%) [95% Confidence Interval]	Sensitivity	Specificity	PPV	NPV	Accuracy	AUC	*p*-Value
Reader 1							
Protocol 1	6/10 (60%) [26–88%]	22/39 (56%) [40–72%]	6/23 (26%) [16–40%]	22/26 (85%) [71–93%]	28/49 (57%) [42–71%]	0.58 [0.43–0.72]	<0.001
Protocol 2	9/10 (90%) [56–100%]	36/39 (92%) [79–98%]	9/12 (75%) [50–90%]	36/37 (97%) [85–100%]	45/49 (92 %) [80–98%]	0.912 [0.80–0.98]	<0.001
Reader 2							
Protocol 1	8/10 (80%) [44–98%]	24/39 (62%) [45–77%]	8/23 (35%) [24–47%]	24/26 (92%) [77–98%]	32/49 (65%) [50–78%]	0.71 [0.56–0.83]	0.03
Protocol 2	9/10 (90%) [56–100%]	37/39 (95%) [83–99%]	9/11 (82%) [54–95%]	37/38 (97%) [85–100%]	46/49 (94%) [83–99%]	0.92 [0.81–0.98]	0.03
Reader 3							
Protocol 1	6/10 (60%) [26–88%]	23/39 (59%) [42–74%]	6/22 (23%) [17–41%]	23/27 (85%) [72–93%]	29/49 (59%) [44–73%]	0.60 [0.45–0.73]	0.04
Protocol 2	8/10 (80%) [44–98%]	36/39 (92%) [79–98%]	8/11 (73%) [46–89%]	36/38 (95%) [84–98%]	44/49 (90%) [78–97%]	0.86 [0.73–0.94]	0.04

Abbreviations: PPV: positive predictive value, NPV: negative predictive value, AUC: area under the curve. Numbers in square brackets are confidence intervals. Diagnostic accuracy of individual readers for protocol 1 (standard grayscale CT) and protocol 2 (standard grayscale CT + color-coded collagen maps) for assessing DTFS injury. MRI or surgical inspection provided the standard of reference.

**Table 4:** Diagnostic confidence, image quality and image noise of standard CT and collagen mapping.

Variables, Mean ± SD [95% Confidence Interval]	Diagnostic Confidence	Image Quality	Image Noise
Grayscale Images	2.3 ± 1.0 [2.1–2.4]	2.4 ± 0.9 [2.2–2.5]	2.3 ± 1.0 [2.1–2.5]
Color-Coded Images	4.1 ± 0.8 [4.0–4.3]	3.9 ± 0.7 [3.8–4.0]	3.6 ± 0.6 [3.5–3.7]
*p*-Value	<0.001	<0.001	<0.001
κ Grayscale	0.33 [−0.06–0.60]	0.32 [−0.07–0.6]	0.36 [−0.03–0.6]
κ Color-Coding	0.71 [0.53–0.83]	0.70 [0.51–0.82]	0.40 [0.03–0.64]

Abbreviations: κ: kappa. Numbers in square brackets are confidence intervals. Diagnostic confidence, image quality, image noise, and Fleiss’ κ of standard grayscale CT and color-coded collagen reconstructions.

## Data Availability

The data presented in this study are available on request from the corresponding author. The data are not publicly available due to data protection guidelines.

## Abbreviations

ATIFL	Anterior Tibiofibular Ligament
AUC	Are Under the Curve
DECT	Dual-Energy Computed Tomography
DTFS	Distal Tibiofibular Syndesmosis
FOV	Field of View
MDCT	Multi-Detector Computed Tomography
NPV	Negative Predictive Value
PPV	Positive Predictive Value
PTIFL	Posterior Tibiofibular Ligament
ROC	Receiver Operator Characteristic
SD	Standard Deviation
